# The Institutional Worker

**Published:** 1918-09-28

**Authors:** 


THE
### INSTITUTIONAL WORKER
**Being a Special Supplement to "The Hospital"**

### OUR BUREAU OF INFORMATION.

### Rules for Correspondents.

1. Every letter must be accompanied by the coupon to be cut from the back cover (inside page) of THE HOSPITAL, current issue, and must contain the name and address of the correspondent with pseudonym for publication if desired. Replies by post cannot be given save under exceptional circumstances at the Editor's discretion.
2. Letters from Approved Homes in reply to special needs published in the Bureau should state terms and full particulars, and be sent prepaid under cover to the Editor of the Bureau with name written across coupon for identification.
3. Proprietors of Homes which have not yet been entered on the List of Approved Homes, but have spare accommodation likely to suit special needs, are invited to write for an application form for registration. The fee for registration, which includes two announcements of the Home in the Bureau and other privileges, is 10s.
4. All communications to be addressed to the Editor of THE HOSPITAL, 28 Southampton Street, Strand, London, W.C. 2, and marked "Bureau of Information."

### INSTITUTION FACTS AND FIGURES.

### Voluntary Workers and the Maternity Act.

THE voluntary worker is tending to become a new type of official. That is to say, while he or she remains voluntary, unsalaried, and unpensionable, and consequently met by no demand for proof of education and handicapped by no regulations as to term of service, he or she is more and more recognised as the link which should not be missing between State or Municipal agency and the public. Work consequently tends to become more definite and more responsible; method, constancy, and punctuality become as important as initiative and freshness. Under the new Maternity and Child Welfare Act at least two women are to be included in the committees required to be set up by every Council working the Act, and the opportunity is a great one for the woman of leisure, education, and experience. Sound and well-thought-out schemes will need to be framed and submitted, and their working carefully watched and guided; new Centres and Homes will be set up and old ones brought into line. Voluntary Associations will receive grants on condition that their work is co-ordinated with that of the Local Authority; they will need in some cases at least more business-like management, and here again the woman who is weary of committees may find that her work, if practical, is anything but in vain.

A number of voluntary workers have done varying proportions of Health Visiting, either in connection with some other object for visits or in following up cases treated at Centres where they helped. Often their visits have done much good, but as the importance of the effort has been more fully recognised the need for the authority derived from proper training has become evident. The appointment of systematic visitors, trained and salaried, has proceeded very fast, and so many will be needed that any woman who can train will be wise to do so. Help towards the expense may be given by Councils. The Local Government Board's grant is extended to make it available for the instruction in hygiene of voluntary as well as salaried workers, if the medical officer thinks such instruction likely to improve their competence. But the voluntary worker who will help at Centres, visit and report to the Centre or to official Health Visitors, and emphatically the one who will work on a Children's Care Committee in any town, is still urgently needed. Some years ago Miss Loane wrote in her clever fashion on the Dark Star who might be a priceless boon to the district nurse if she would live with her, cheer her times of meals and rest, and keep home affairs going, while helping her actual work by preparation of appliances, and such services as cheering visits to tedious cases. Volunteer workers should read the letter to Councils entitled "Maternity and Child Welfare" (summarised in THE HOSPITAL of August 31, p. 480), giving the new regulations and many suggestions which the President of the Local Government Board has sent out. It costs 1d. from any bookseller, or from H.M. Stationery Office, Kingsway, London, W.C. 2, and elsewhere.

### The Milk Bill in a Small Fever Hospital.

"BANFFSHIRE" asks our readers' opinions on the question of the milk consumption in a small fever hospital and generally on the menu. Do they consider that the amount of milk used is excessive or otherwise, and the menu adequate or the reverse? These are practical points concerning which the experience of institution administrators and managers cannot fail to be of value. The question as to the milk is of special importance.

The hospital is open for three infectious diseases only—scarlet-fever, typhoid, and diphtheria.

Last year fifty-one cases were admitted into hospital, of which one was an adult typhoid who was fond of milk and had large quantities. The staff consisted of six adults. The quantity of milk consumed was 994½ gallons, costing £88 3s. 9½d.

MENU FOR CONVALESCENT PATIENTS.

*Breakfast.*—Porridge and milk, bread, margarine, syrup. Tea for adults.

*Dinner.*—Fish (once a week), mince (twice weekly), soup (daily), Scotch broth, rice soup, pea and lentil hough, etc. Milk pudding (daily), viz.: rice, ground rice, tapioca, large and small sago, semolina, cornflour, etc., in rotation. Adults: Butchers' meat four times weekly.

*Tea.*—Bread, margarine, jam or jelly, and syrup.

*Supper.*—Milk pudding, bread, margarine, and syrup. Adults: Cocoa, gruel, or milk.

IN CONVALESCENT CASES AFTER SEVERE ILLNESS.

*Breakfast.*—Eggs, fish, bacon.

*Dinner.*—Chicken, meat, fish, rabbit, etc., and butter instead of margarine.

The matron superintends the cooking so that there is no waste, and the patients are not over-restricted in the amount of food they may have.

### SEPTEMBER QUESTION.

### Fuel and Lighting Economies.

**1. Describe means by which economies may be effected in the consumption of fuel and lighting in an institution (a) in the wards, ward kitchens, etc., (b) in the kitchens and servants' quarters, (c) in the Nurses' Home, (d) in the administration building.**

**2. What steps may be taken in the engineer's department to keep up the heating and lighting and hot-water supplies with the least possible expenditure of fuel?**

**(A minimum payment of 10s. 6d. will be made for each published answer. It is not imperative for both parts of the question to be answered.)**

#### RULES.

The following rules must be observed:—

1. Contributions must be written on one side of the paper. Brevity and terseness are desirable features. The MSS. must bear the name and address of the sender and be accompanied by coupon to be cut from the back cover (inside page) of the current issue of THE HOSPITAL. A pseudonym must be chosen if the name is not to be published.

2. Contributions must be addressed to the Editor of THE HOSPITAL, Institutional Worker Supplement, 28 & 29 Southampton Street, Strand, London, W.C. 2, should reach him before the end of the current month, and be marked in the left-hand corner "Facts and Figures."

### QUESTIONS INVITED.

IN connection with our Question Box, we offer every month five shillings each for the two best questions which are sent in for consideration. The questions may be on any institutional subject and concern any department, from the secretary's to the porter's, or the matron's to the domestic staff; the one essential is that they must be practical and deal with points that have a definite relation to hospital or institutional administration, upkeep, finance, or management. Points relating to artificers' work, housekeeping, the laundry, out-patients, and other practical matters will be welcome. It is hoped that thus, when difficult or doubtful points arise in the course of their work, institutional workers will be encouraged to put them in the form of a question and send them to the Editor, THE HOSPITAL Bureau of Information, 28 & 29 Southampton Street, Strand, W.C. 2, marked "Question Box."

The best questions will be published in due course and our readers will be given the opportunity of answering them. Thus all institutional workers may be able to cooperate with the view of helping the work and smoothing the difficulties of each other. In short, we want the Question Box of the *Institutional Worker* to be freely used by every worker habitually.

### ENQUIRIES AND ANSWERS.

### *EMPLOYMENT AND TRAINING.*

### Midwifery Training in Dublin.

APPLICANTS are received at the Coombe Lying-in Hospital and Guinness Dispensary, Dublin, for six months' midwifery training. The premium for resident pupils is £18 18s.; for non-resident, £6 6s. Registration fee 10s. 6d. There is a course of one month for gynæcological diploma—fee £2 2s. A candidate for the English C.M.B. examination is required to pay an extra fee of £3 3s.—SHAMROCK.

### Infirmary Training-school in Birmingham.

THERE are three large infirmary training-schools in Birmingham. The Infirmary, Birmingham, receives suitable applicants for a course of three year's training between the ages of twenty-one and thirty-three. At the Workhouse Infirmary, Erdington, Birmingham, a three years' course is given; a limited number of probationers are chosen to enter the maternity wards for a course of instruction in midwifery to qualify for the examination of the C.M.B. Nurses availing themselves of this privilege are required to remain an extra nine months. Three years' training is also given at Birmingham Union Selly Oak Infirmary. At this institution also an extra nine months' training is necessary to qualify for the C.M.B. examination. All these institutions are training-schools recognised by the Central Midwives Board.—STREETLY.

### *THE HOUSEKEEPERS' DEPARTMENT.*

### Queen of Puddings.

THE following makes an excellent and not expensive pudding. You will require 1 pint of breadcrumbs, 1 pint of milk, half a cupful of sugar (or less if desired), grated rind of lemon, 2 tablespoonfuls of egg powder. Beat the whole well together and place it in a well-greased dish and bake in a slow oven until quite set. Take it out of the oven and spread a thin layer of jam over the top; beat the white of one egg, and spread lightly over the jam; put back into the oven and bake a light brown. This makes sufficient for five persons.—S. E. L.

### Soap and Caustic Mixture for Laundries.

THE usual average mixture of soap and caustic soda for use in the laundry is 25 lbs. of soap to 100 gallons of water, with the addition of 3 lbs. of caustic. In place of the caustic, 10 lbs. of ordinary washing soda (carbonate of soda) can be used; in fact, this mixture is preferred by some.—TONY.

### *MISCELLANEOUS.*

### Address Wanted.

THE address of the Scottish Branch of the College of Nursing, Ltd., is 122 George Street, Edinburgh.—FAR NORTH.

### A Dog's Olfactory Organ.

IT does not come within the province of these columns to give information upon matters of the character referred to by you. Your proper course is to consult a veterinary surgeon.—STUDENT.

### A Useful Cement.

AN excellent plastic repair compound known as "Farotex" can be obtained from S. and W. Farmiloe, Ltd., of Rochester Row, S.W. 1. It is especially useful for stopping cracks in flat roofs, and it will adhere to any surface, whether tin, zinc, lead, slate, tile, or glass.—G. A. G.

### Cinder Sifter and Dust-bin Combined.

MESSRS. FREDK. BRABY AND CO., LTD., Fitzroy Works, 352 to 364 Euston Road, N.W., supply an excellent galvanised automatic cinder sifter and dust-bin combined. It is both sanitary and economical. It absolutely prevents waste and saves both time and labour. The cinder and refuse drawers are specially constructed to allow of ready removal and replacement. The combined article is on four wheels and is easily moved from place to place. The large size measures 1 ft. 8 in. wide by 2 ft. deep (front to back) by 3 ft. 2 in. high at back (including wheels). The price is £3 7s. 6d.—COAL ECONOMIST.

### "Water-fuel" Furnaces.

YOU have doubtless been misled in this matter. The use of the somewhat loose term "water fuel" requires explanation. Water alone will not provide heat for a furnace; it can be used in combination with oil, petrol, or the heavier products of coal such as tar. The use of water to aid combustion is based upon the fact that when passed through a very hot metal coil the water not only vaporises, but is split up into the constituent elements—oxygen and hydrogen. In order to heat the furnace oil is first lighted and the flame from it passes up through a coil of copper tube, making it red-hot; water is then turned into the hot coil, where it is split up and the oxygen and hydrogen gases pass into the oil flame. The hydrogen burns with the hydro-carbons of which the oil is composed, and combustion is aided with great intensity by the freed oxygen.—NOVICE.

**The Editor will be glad to receive correspondence and to consider contributions upon all subjects relating to institutional work which affect the welfare of institutional workers.**

